# New Phage-Derived Antibacterial Enzyme PolaR Targeting *Rothia* spp.

**DOI:** 10.3390/cells12151997

**Published:** 2023-08-04

**Authors:** Paulina Miernikiewicz, Jakub Barylski, Aleksandra Wilczak, Anna Dragoš, Izabela Rybicka, Sophia Bałdysz, Aleksander Szymczak, Iztok Dogsa, Kostiantyn Rokush, Marek Adam Harhala, Jarosław Ciekot, Stanisław Ferenc, Jan Gnus, Wojciech Witkiewicz, Krystyna Dąbrowska

**Affiliations:** 1 Hirszfeld Institute of Immunology and Experimental Therapy, Polish Academy of Sciences, 53-114 Wrocław, Poland; aleksandra.wilczak@hirszfeld.pl (A.W.); izabela.rybicka@hirszfeld.pl (I.R.); aleksander.szymczak@hirszfeld.pl (A.S.); kostiantyn.rokush@hirszfeld.pl (K.R.); marek.harhala@hirszfeld.pl (M.A.H.); jaroslaw.ciekot@hirszfeld.pl (J.C.); dabrowska@hirszfeld.pl (K.D.); 2 Department of Molecular Virology, Faculty of Biology, Adam Mickiewicz University, 61-712 Poznań, Poland; jakub.barylski@amu.edu.pl (J.B.); sophia.baldysz@amu.edu.pl (S.B.); 3 Department of Microbiology, Biotechnical Faculty, University of Ljubljana, 1000 Ljubljana, Slovenia; anna.dragos@bf.uni-lj.si (A.D.); iztok.dogsa@bf.uni-lj.si (I.D.); 4 Research and Development Center, Regional Specialist Hospital in Wrocław, 51-124 Wrocław, Poland; ferenc1@o2.pl (S.F.); jan.gnus@umw.edu.pl (J.G.); wojciech.witkiewicz@wssk.wroc.pl (W.W.); 5 Faculty of Health Sciences, Wrocław Medical University, 50-367 Wrocław, Poland

**Keywords:** endolysin, bacteriophages, *Rothia*, phageome, stomach, bacteriolytic

## Abstract

*Rothia* is an opportunistic pathogen, particularly life-threatening for the immunocompromised. It is associated with pneumonia, endocarditis, peritonitis and many other serious infections, including septicemia. Of note, *Rothia mucilaginousa* produces metabolites that support and increase overgrowth of *Pseudomonas aeruginosa*, one of the ESKAPE bacteria. Endolysins are considered as antibacterial enzymes derived from bacteriophages that selectively and efficiently kill susceptible bacteria without harming human cells or the normal microbiome. Here, we applied a computational analysis of metagenomic sequencing data of the gastric mucosa phageome extracted from human patients’ stomach biopsies. A selected candidate anti-*Rothia* sequence was produced in an expression system, purified and confirmed as a *Rothia mucilaginosa*- and *Rothia dentocariosa*-specific endolysin PolaR, able to destroy bacterial cells even when aggregated, as in a biofilm. PolaR had no cytotoxic or antiproliferative effects on mammalian cells. PolaR is the first described endolysin selectively targeting *Rothia* species, with a high potential to combat infections caused by *Rothia mucilaginosa* and *Rothia dentocariosa*, and possibly other bacterial groups. PolaR is the first antibacterial enzyme selected from the gastric mucosa phageome, which underlines the biological complexity and probably underestimated biological role of the phageome in the human gastric mucosa.

## 1. Introduction

*Rothia* spp. are Gram-positive, non-motile, non-sporogenic, aerobic or facultative anaerobic, encapsulated bacteria belonging to the phylum Actinobacteria [1,2]. In 1967, George and Brown first described the *Rothia* genus [3] to accommodate the earlier known organisms *Actinomyces dentocariosus* [4] and *Nocardia salivae* [5]. To date, 15 species of *Rothia* have been identified [6,7]. Five species within the genus *Rothia* are human pathogens: *Rothia mucilaginosa*, *Rothia dentocariosa*, *Rothia aeria*, *Rothia kristinae* and *Rothia koreensis* [1,2,8,9]. *Rothia mucilaginosa* and *Rothia dentocariosa* colonize the human oral cavity and upper and lower respiratory tract [2,7], while *Rothia kristinae* is commonly found on human skin [10]. *Rothia* is considered an opportunistic pathogen particularly in immunocompromised patients [11,12,13,14]. It is associated with a wide range of diseases including endocarditis, pneumonia, peritonitis and septicemia. *Rothia* has been described as the causative agent of abdominal infections, tonsillitis, spondylodiscitis, keratitis, meningitis, osteomyelitis, bronchitis and infections related to catheters and prosthetic devices, as well as infections of peritoneal fluid, sputum, synovial fluid and bile [1,2,8]. The presence and metabolic activity of *Rothia mucilaginosa* have been identified in the lungs of cystic fibrosis (CF) patients [15,16,17,18,19]. Moreover, *Rothia mucilaginosa* has some features that may influence the severity and progression of chronic respiratory disorders, especially in CF patients. *Rothia mucilaginosa* generates metabolites that support *Pseudomonas aeruginosa* growth, thus establishing a *Pseudomonas aeruginosa*-promoting environment [16,18]. Further, *Rothia mucilaginosa* forms biofilms similar to *Pseudomonas aeruginosa* and may mitigate inflammation in the lower airways; thus, it may promote colonization and survival of this species in the lung environment [20,21]. *Rothia* infections are not common in clinical practice, but their real number is likely underestimated. Possible reasons for this are the common view of *Rothia* as part of the normal human microbiota in clinical laboratories and its misidentification by phenotypic characterization [2,12].

Endolysins, bacteriophage-encoded enzymes capable of degrading peptidoglycan and thus killing bacteria, are one of the promising new antibacterial agents. Notably, they are considered a promising alternative to antibiotics [22], due to a very different mode of action and no bacterial cross-resistance observed. Successful use of endolysins against MRSA, *Listeria monocytogenes*, *Staphylococcus* or *Pseudomonas aeruginosa* strains, including those forming biofilms, has inspired increasing interest in these enzymes [23,24,25,26]. To date, no endolysin specific for any *Rothia* species has been identified. 

Here, we present, for the first time, the new endolysin PolaR, specific to *Rothia* spp. The gene coding for PolaR was found in stomach virome metagenomes, obtained from human gastric biopsies. In this study, we characterized the genomic and evolutionary background of the gene encoding PolaR, predicted the structure of the enzyme and analyzed its antibacterial activity. Due to the safety requirements for human or animal treatments, general safety testing on mammalian cells (cytotoxicity testing) has also been completed.

## 2. Materials and Methods

### 2.1. Biological Sample Collection

The stomach biopsies were collected from adult patients during endoscopy examination in the Endoscopy Department of the Regional Specialist Hospital in Wroclaw. The collections were made in accordance with the ethical standards of the Helsinki Declaration and were approved by the Local Bioethical Committee in the Research and Development Center, Regional Specialist Hospital in Wroclaw (no. KB/nr 8/rok 2017). Endoscopy procedures were conducted by qualified surgeons Jan Gnus, MD PhD and Stanisław Ferenc, MD PhD. Samples were collected between 18 September 2018 and 2 October 2019.

### 2.2. DNA Extraction

DNA extraction from clinical samples was conducted according to [27] (with some modifications). Briefly, each biopsy was shaken on a 3D shaker for 3 h at 4 °C and centrifuged at 12,000× *g* for 10 min. The supernatant was filtered through a 0.22 μm pore membrane and loaded on a CsCl gradient (density layers of 1.7, 1.5, 1.35 and 1.15 g mL^−1^) for virome separation. Cesium chloride centrifugation was performed at 62,000× *g*, at 4 °C for 22–24 h. The obtained virome was located between the density fractions of 1.5 and 1.35 g mL^−1^ and withdrawn for the further procedure. Virome DNA isolation (Sherlock AX kit, A&A Biotechnology, Gdańsk, Poland) was carried out and samples with minimum DNA concentrations of 1 ng/µL were amplified (Genomiphi V2 DNA Amplification kit, Cytiva, Marlborough, MA, USA). To prepare sequencing libraries, Illumina DNA Prep with Nextera DNA CD indexes (Illumina, San Diego, CA, USA) was used. The virome DNA concentration was estimated using a Quantus Fluorometer with the QuantiFluor dsDNA system (Promega, Walldorf, Germany).

### 2.3. Illumina Sequencing and Processing of Sequencing Data

Libraries were pulled by combining an equal mass of each library and sequenced using the NextSeq 500/550 Mid Output Kit v2.5 (300 Cycles) on the NextSeq550 instrument. The obtained data were transformed into FASTQ files by BaseSpace (Illumina). The whole phageome sequencing data obtained in this project are available at NCBI BioProject (acc. No. PRJNA934363), while the specific sample that was the source of the PolaR was submitted as BioSample SAMN33273746 corresponding to SRA record SRX19355672.

### 2.4. Assembly of Metagenomic Contigs

Viral metagenomes (viromes) were assembled using metaSPAdes v3.15.2 [28]. Prior to assembly, reads were trimmed using Trimmomatic v0.39 [29]. For each sample, we performed two independent analyses: one based on strict (SLIDINGWINDOW:5:30) and the other on relaxed (SLIDINGWINDOW:5:25) trimming settings. In both cases, we used the ILLUMINACLIP step to remove the remains of the adapter sequences and discard reads shorter than 20 bp. All of the generated contigs were used to search for potential lytic proteins in the downstream procedures.

### 2.5. Identification of Genes Coding New Antibacterial Enzymes

The assembled contigs were scanned for phage-like sequences using the Vibrant v1.2.1 (Virus Identification By iterative ANnoTation) [30], which included the PFAM 32.0 and pVOG 94 databases. Results of this pipeline include preliminary gene annotations (based on the Prodigal prediction tool configured for metagenomic analysis and HMMer3 search against the Pfam and VOG databases); these annotations were used to select genes encoding potential lysin sequences. Candidate endolysins were selected from these proteins by comparison with an in-house lysin-related HMM database and manual curation. Sequences of interest were de-replicated by clustering with the CD-hit 4.7 algorithm at 75% sequence similarity [31]. The final choice of the candidates for cloning was based on the assessment of possible targets of the putative lysin. We analyzed putative hosts of the phages represented by the contigs encoding the predicted lysins by a PHIST 1.1.0 search against 17546 non-redundant bacterial genomes from the RefSeq database [32]. Contigs with top hits against pathogenic bacteria were manually curated based on the results of BLASTn, BLASTx and/or tBLASTx searches against the entire RefSeq database [33] and the Vibrant annotations.

### 2.6. Analysis of the Genomic and Evolutionary Background of the Selected Lysin

To understand the origin of the selected lysin sequence, we performed an in-depth analysis of the evolutionary and genomic context of the gene encoding PolaR. First, 50 reference protein sequences most similar to the enzyme were retrieved by a BLASTp search (word size 6, other settings default) against the RefSeq protein database. These sequences were aligned using the Clustal Omega used to create a phylogenetic tree with the FastTree plugin from the same software package (both run with default settings). Visualization of the resulting tree was performed using Geneious Prime^®^ v.2022.1 (Biomatters, Inc., Auckland, New Zealand). Alignment and tree-generating programs were also run as plugins from this software suite. We searched for similar proteins from metagenomic sequences in the IMG/VR (v4) using the provided BLASTp tool [34]. The contigs encoding these proteins were retrieved by searching the “all_nucleotides-high_confidence.fna” file from IMG_VR_2022-09-20_6.1 for scaffold IDs matched to hit proteins using the “Sequence_information-high_confidence.tsv” table.

### 2.7. Prediction of Enzyme Structure

The domain architecture of the selected enzyme was determined using the InterProScan plugin in the Geneious Prime^®^ v.2022.1 software [35]. The 3D structure of the PolaR lysin was predicted using AlphaFold2 available through the ColabFold v1.5.2 online service (https://colab.research.google.com/github/sokrypton/ColabFold/blob/main/AlphaFold2.ipynb, accessed on 21–24 April 2023) [36]. We predicted monomeric, dimeric and trimeric structures and estimated the possibility of multimerization by assessing pLDDT, pTM and ipTM statistics of obtained structures. In all cases, mmseqs2 alignments, fold prediction and preliminary visualization were run using the provided pipeline with default parameters and databases (accessed 24 April 2023). The resulting structures were visualized using UCSF ChimeraX [37]. The secondary structure of the protein was estimated based on coordinates from AlphaFold2 PDB files using DSSP server (http://bioinformatica.isa.cnr.it/SUSAN/DSSP-web accessed on 28 July 2023) [38]. Top-scoring monomeric and dimeric structures (in terms pLDDT) and related metadata were submitted to ModelArchive (https://modelarchive.org) as records ma-ibu1l and ma-8fuis.

### 2.8. Protein Production

The gene coding PolaR was synthesized de novo and cloned into the pBADHisA plasmid using *Nco*I and *Xho*I restriction enzymes. The resulting vector included the gene *PolaR* with the C-terminal 6xHis-tag (BioCat GmbH, Heidelberg, Germany).

Expression was conducted in *Escherichia coli* C43(DE3) at 37 °C with shaking in Luria-Bertani (LB) broth (10 g/L tryptone, 10 g/L NaCl, 5 g/L yeast extract) supplemented with ampicillin (50 mg/L) until OD_600_ = 1.0–1.2. Then, protein expression was induced by the addition of L-arabinose at a final concentration of 2.5 g/L and the culture was incubated for 3 h at 30 °C with intensive shaking. Bacteria were harvested (7000 g, 5 min, 4 °C) and the pellet was suspended in phosphate buffer (50 mM NaH_2_PO_4_ × H_2_O, 300 mM NaCl, pH = 8.0) supplemented with an inhibitor of serine protease (PMSF (1 mM)) and lysozyme (1.5 mg/mL). The bacterial suspension was incubated for 6–7 h on ice with gentle shaking and lysed using the freeze–thaw method. DNase (up to 30 μg/mL) with Mg^2+^ (up to 1 mM) and RNase (up to 60 μg/mL) was then added to the extract and incubated on ice for 3 h with gentle shaking. The fractions were separated by centrifugation (12,000× *g*, 30 min, 4 °C). The soluble fraction was collected and filtered sequentially through 0.45 µm and 0.22 µm PES sterile filters. Using NiNTA agarose (Thermo Fisher Scientific, Waltham, MA, USA), 6 × His-tagged PolaR was purified by affinity chromatography. The cleared supernatant was supplemented with 10 mM imidazole and mixed with NiNTA agarose at room temperature followed by washing steps with phosphate buffer supplemented with an increasing concentration of imidazole: 25 mM, 50 mM, 75 mM, 100 mM, 250 mM and 500 mM at pH = 8. Fractions with high protein concentrations were dialyzed against PBS at 4 °C, concentrated with Vivaspin and further purified using size exclusion chromatography on a Superdex 75 10/300 GL column (GE Healthcare Life Sciences, Chicago, IL, USA). The final step was LPS removal, which was performed with the endotoxin removal resin, EndoTrap^®^ HD (LIONEX GmbH, Braunschweig, Germany). The LPS level was checked using ENDOZYME II (BioMerieux, Marcy_l’ Étoile, France) according to the manufacturer. Purified, endotoxin-free protein samples were dialyzed against PBS and filtered through sterile 0.22 μm PES filters. The protein concentration was determined spectrophotometrically.

### 2.9. PolaR’s Lytic Activity—Fluorometric Reduction Assay

The bacteriolytic activity of PolaR was determined by a fluorometric assay using Sytox Green nucleic acid stain (Thermo Fisher Scientific, Waltham, MA, USA) according to [39] (with some modifications). Five *Rothia* strains were subjected to the activity test: *Rothia dentocariosa* PCM 2348 (ATCC 14189), *Rothia dentocariosa* PCM 2349 (ATCC 17931), *Rothia mucilaginosa* PCM 2403, *Rothia mucilaginosa* PCM 2415 (ATCC 25296) and *Rothia aeria* PCM 2669.

Briefly, overnight liquid cultures (brain–heart infusion broth (BHI, Oxoid)) of *Rothia* strains were inoculated in fresh BHI medium and cultivated to the late log phase at 37 °C with shaking. All bacteria were harvested by centrifugation (7000× *g*, 7 min, 4 °C) and washed twice in PBS. The last pellet was resuspended in PBS to the final OD_600_ of 0.6. Samples for the Sytox Green assay were prepared as follows: 130 μL of OD_600_ = 0.6 of bacterial suspension, 20 µL of PolaR preparation (at final concentration 1, 25, 100 µg/mL) or PBS, 50 µL of diluted (1/1000) Sytox Green stain. All samples were tested in triplicate. Kinetic tests were performed in 96-well black plates for 9–24 h at room temperature in the fluorescence reader. Fluorometric assays were conducted 3 times on three independently produced PolaR preparations.

### 2.10. Antibacterial Activity Test Using Single Cell Microscopy

*Rothia mucilaginosa* PCM 2415 (ATCC 25296) was prepared in the same way as described in the section ‘Antibacterial activity—fluorometric assay’. An amount of 1 mL of bacterial suspension in PBS with OD_600_ = 0.6 was stationary incubated overnight at room temperature with 150 µg/mL of PolaR at the final concentration or with PBS. The LIVE/DEAD^®^ BacLight^TM^ Bacterial Viability Kit (Invitrogen, Waltham, MA, USA) was used for microscopy and quantitative analysis of aggregates according to the manufacturer. Images were acquired using the differential interference contrast (DIC) technique as well as fluorescence microscopy. Fluorescence images were acquired by an inverted confocal laser scanning 487 microscope (AxioVision Z1, LSM800) (Zeiss, Germany) using the EC Plan-Neofluar 100×/1.40 oil Plan apochromat objective and two laser channels: a 488 nm laser to acquire green fluorescence and a 561 nm laser to acquire red fluorescence.

### 2.11. Antibacterial Aggregate Activity—Fluorometric and Turbidity Reduction Assay

*Rothia mucilaginosa* PCM 2415 (ATCC 25296) was prepared in the same way as described in the section ‘Antibacterial activity—fluorometric assay’. Part of the bacterial suspension was gently sonicated for 15 s to avoid bacterial cell destruction. The viability of the sonicated bacterial suspension was monitored under a fluorescence microscope using the LIVE/DEAD^®^ BacLight^TM^ Bacterial Viability Kit (Invitrogen, Waltham, MA, USA) and only living sonicated cells were further tested. Samples for the assay were prepared as follows: 130 µL of bacterial suspension in PBS (OD_600_ = 0.6), 20 µL of PolaR preparation at a final concentration of 150 µg/mL or PBS and 50 µL of propidium iodide stain. Fluorescence measurement was conducted simultaneously with OD_600_ readings (turbidity reduction assay, TRA) in triplicate. Kinetic tests were performed in 96-well black plates with a clear bottom for 16 h at room temperature in the fluorescence reader.

### 2.12. Safety Assay of PolaR

The potential cytotoxicity of PolaR was assessed by an MTT (3-[4,5-dimethylthiazol-2-yl]-2,5 diphenyl tetrazolium bromide) assay according to [40]. Three cell lines were investigated: FaDu (ATCC HTB-43) human pharynx squamous cell carcinoma, RAW 264.7 (ATCC TIB-71) mouse monocyte/macrophage and A549 (ATCC CCL-185) human lung adenocarcinoma. FaDu cells were cultured in RPMI 1640 (Gibco) medium, RAW 264.7 cells in DMEM (Gibco) medium and A549 cells in DMEM/F12. A total of 10⁴ cells were seeded per well in 90 µL of medium in a 96-well plate and grown overnight. Next, 10 µL of PolaR solution in PBS was added to the wells to achieve 0.1, 1, 10 and 100 µg/mL final concentrations and incubated with cells for 48 h. PBS-treated cells were used as a control. Then, 10 µL of MTT reagent was added to each well and after 4 h, 50 µL of detergent solution (1M SDS in 45% DMF) was added to each well. After 24 h, the absorbance was measured at 570 nm on the microplate reader. All samples were tested in 8–10 replicates. The results were presented as a percentage of the control (non-treated cells) signal.

## 3. Results

### 3.1. Investigating Human Stomach Phageomes for the Selection of Candidate Endolysins

In this study, stomach phageomes from patients affected by gastric disorders were used to find new enzymes with antibacterial potential, particularly those targeting *Rothia* spp. The stomach phageome metagenome, obtained from the gastric biopsies and containing the mucosal fraction, was sequenced. Reads resulting from sequencing of each of the 60 libraries were assembled (Supplementary Materials File S1). Depending on the trimming procedure, we assembled 7–12 million contigs with a total length of 2–5 Gb, respectively. The number of viral contigs recovered from all samples was 749–1556, corresponding to ~0.3% of the total contig length (such a low number is probably a result of discarding numerous short, uninformative sequences of <1000 bp prior to the Vibrant classification, for details see Supplementary Materials File S1). A total of 90 non-redundant contigs from both assemblies were annotated as lysin-containing ones. After careful inspection of hosts predicted for these contigs by PHIST and re-evaluation of selected predictions by BLAST, we selected an endolysin gene for further investigation. The chosen sequence was found in the virome from the stomach biopsies collected from a 71-year-old female patient suffering from duodenal bulbitis and chronic gastritis (SRA record SRX19355672, biosample SAMN33273746, grouped with other libraries from this study under BioProject accession number PRJNA934363). This selected sequence encoded the putative protein (herein named PolaR), composed of 193 amino acids, that had a theoretical weight of 21.8 kDa and an isoelectric point of 4.55. The sequence carried the HMM signatures of the peptidoglycan recognition protein and N-acetylmuramoyl-L-alanine amidase, which usually bind and hydrolyze peptidoglycans of Gram-positive bacteria.

**Figure 1:**
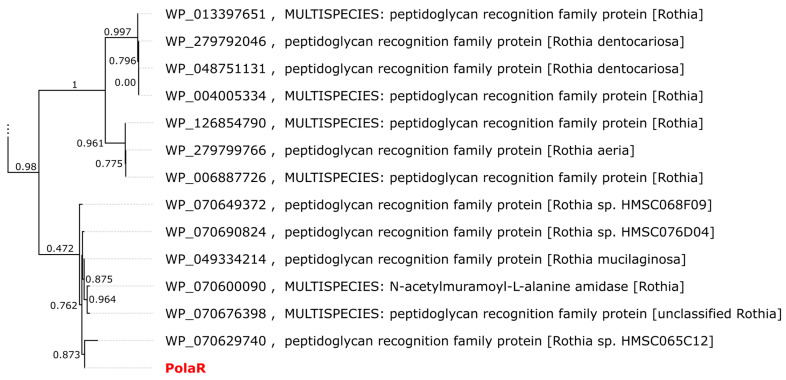
Approximate maximum likelihood (ML) tree of proteins similar to PolaR retrieved from the RefSeq database. The dendrogram was calculated using FastTree 2.1.11 with the Jones–Taylor–Thornton (JTT) model. Branch labels represent support from the Shimodaira–Hasegawa test. Only the two clades closest to PolaR are shown. The complete tree is available as newick/nexus in Supplementary Materials File S1.

While the top BLAST hits for the PolaR sequence are annotated as proteins from actinobacteria (Figure 1), Vibrant classified the contig encoding the lysin as a phage sequence.

A search against the IMG/VR (v4) database, which contains millions of viral genomes and genome fragments assembled during metagenomic analyses, revealed 146 similar proteins, each encoded on a separate contig. These sequences were predominantly isolated from animal-associated biomes. The vast majority (117) came from the human oral cavity, but a few were found in the pharynx (or throat), skin, tonsils, intestine and, finally, stomach. Twelve fragments were assembled from other types of environments including marine ecosystems, active sludge and a plant rhizosphere.

**Figure 2:**
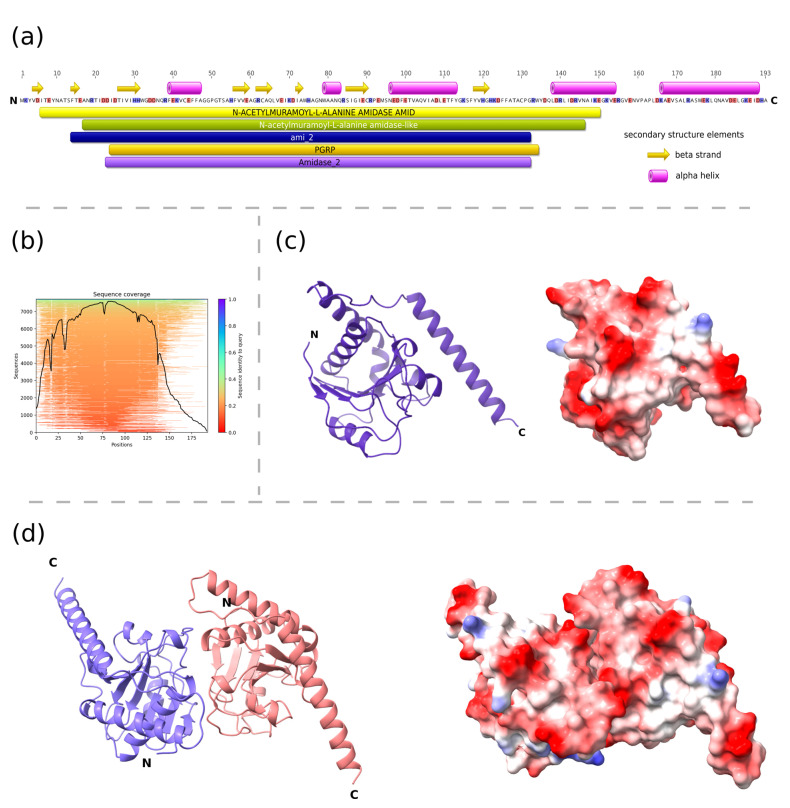
Structural characteristics of PolaR endolysin. (**a**) The domain composition of the lysin was predicted using the InterProScan tool and elements of the secondary structures were assigned based on the AlphaFold2 structure of the monomer using the DSSP method, (**b**) MMseqs2 MSA coverage plot that shows similarity to potential templates located in the ColabFold databases, (**c**,**d**) three-dimensional models of the monomer and putative dimer of PolaR were predicted by AlphaFold2 and visualized using ChimeraX. The ribbon representations are colored according to the polypeptide chain. Residues on the surface representations are colored based on their charge, ranging from red (positive) to blue (negative). Letter “N” and “C” on panels (**a**,**c**,**d**), represents amino-terminus and carboxyl-terminus of the protein respectively.

The predicted structure of PolaR consists of an N-terminal globular N-acetylmuramoyl-L-alanine amidase domain and a free C-terminal helix (Figure 2). The relatively high confidence metrics of the two top-scoring dimeric structures (pLDDT > 87 pTM > 0.8 and ipTM > 0.7) suggest that the protein may form dimers, which was also confirmed further on native SDS-PAGE (Figure S4).

### 3.2. Antibacterial Activity of the New Candidate Endolysin

**Figure 3:**
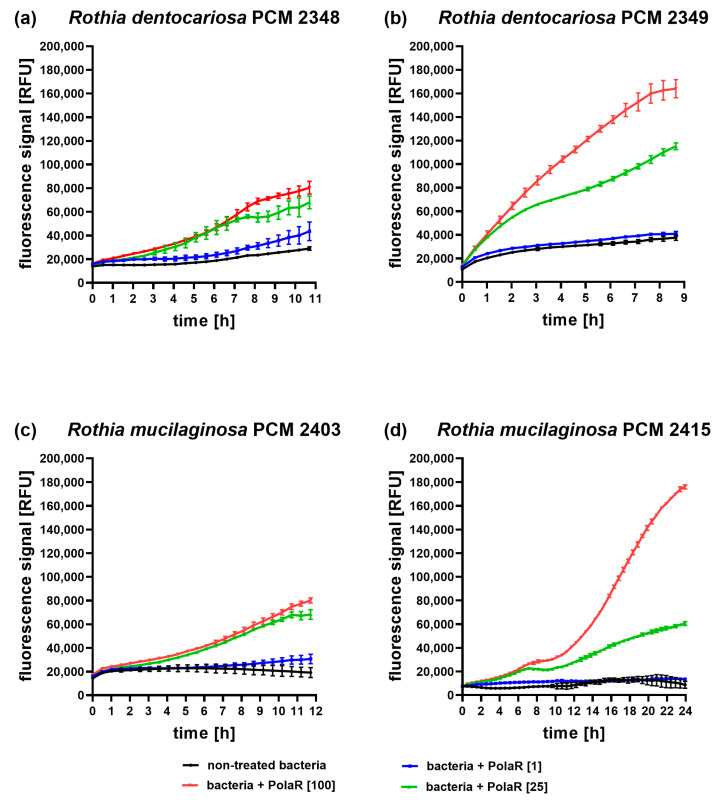
Antibacterial activity of PolaR against *Rothia* spp. over time. (**a**) *Rothia dentocariosa* PCM 2348 (ATCC 14189), (**b**) *Rothia dentocariosa* PCM 2349 (ATCC 17931), (**c**) *Rothia mucilaginosa* PCM 2403 and (**d**) *Rothia mucilaginosa* PCM 2415 (ATCC 25296) were tested in the Sytox Green fluorometric assay with three different PolaR concentrations: 1, 25 and 100 µg/mL. Increasing fluorescence represents an increasing number of killed bacterial cells. All samples were tested in triplicate. The presented data represent one of three independently conducted experiments on three independently produced PolaR preparations.

The gene selected as coding the potential new endolysin capable of targeting *Rothia* spp. was synthesized de novo, cloned into an expression vector and effectively produced (Figure S1). The antibacterial activity of the new candidate endolysin was investigated on five *Rothia* strains. PolaR proved to be active against four (out of five tested) bacterial strains; it was able to lyse *Rothia mucilaginosa* PCM 2403, *Rothia mucilaginosa* PCM 2415 (ATCC 25296), *Rothia dentocariosa* PCM 2348 (ATCC 14189) and *Rothia dentocariosa* PCM 2349 (ATCC 17931), but was not active against *Rothia aeria* PCM 2669 (Figure 3 and Figure S2). Lytic activity was dose dependent within tested concentrations (1, 25, and 100 µg/mL). Notably, complete lysis of the bacteria was observed within approximately 9 h, which is markedly slower than in other endolysins (anti-streptococcal Pal and Cpl-1) investigated in similar conditions [39].

### 3.3. Antibacterial Aggregate Activity of the New Candidate Endolysin

**Figure 4:**
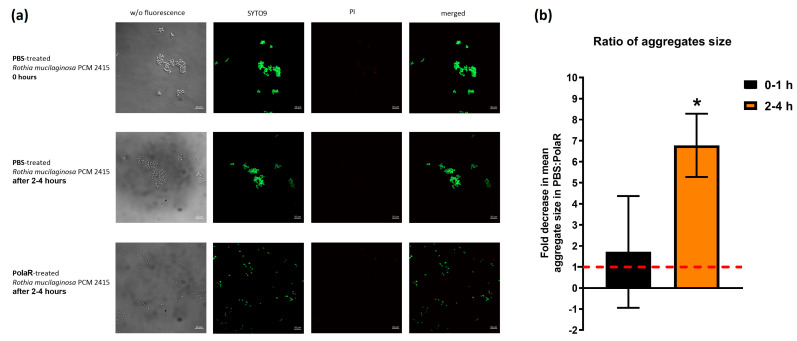
The effect of PolaR on *Rothia mucilaginosa* PCM 2415 (ATCC 25296) aggregates. (**a**) LIVE/DEAD staining of *Rothia mucilaginosa* PCM 2415 (ATCC 25296) after 2–4 h treatment with PBS or 150 µg/mL of PolaR. (**b**) Ratio of aggregate size in 2–4 h after PBS or 150 µg/mL PolaR treatment. PBS fold change is marked as a red dashed line (* *p* = 0.02).

*Rothia mucilaginosa* is an encapsulated bacterium that forms aggregates and produces a robust biofilm [2,7,41,42]. Since many endolysins demonstrate efficient activity against biofilms [25,43,44,45,46,47], the new endolysin PolaR was studied for its capability to target *Rothia* aggregates. Fluorescence microscopy revealed that the investigated *Rothia* strain formed aggregates with a mean size of 11 µm^2^ (Figure 4a,b).

**Figure 5:**
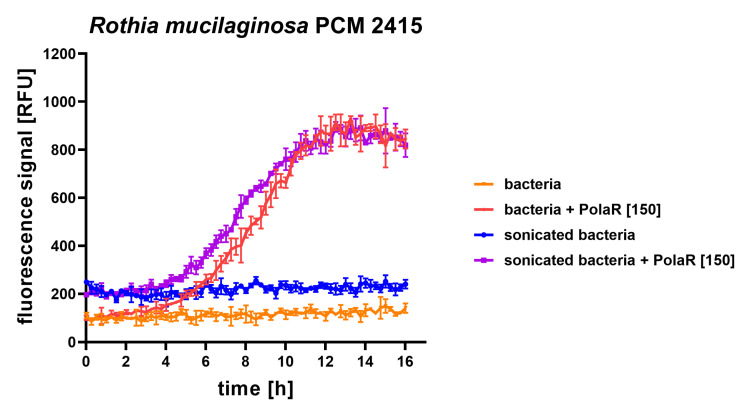
Antibacterial activity of 150 µg/mL PolaR against sonicated and non-sonicated *Rothia mucilaginosa* PCM 2415 (ATCC 25296) was tested over time in a fluorometric assay. Increasing fluorescence represents an increasing number of killed bacterial cells.

In 2–4 h of PolaR treatment, Rothia aggregates were disrupted and appeared as single cells. Some dead bacterial cells were also visible (Figure 4a). The mode of PolaR action was also verified on preliminary disrupted aggregates of *Rothia* cells. Surprisingly, gently sonicated *Rothia* cells revealed only minor and insignificant differences in their sensitivity to PolaR compared to non-sonicated cells. These data were observed in the fluorometric assay (Figure 5) and TRA (Figure S3). All these results indicate that PolaR is fully active against aggregated bacteria, acts against bacterial clumping and disrupts aggregated bacterial cells, efficiently killing bacteria. 

### 3.4. Safety of PolaR Testing on Mammalian Cell Cultures

**Figure 6:**
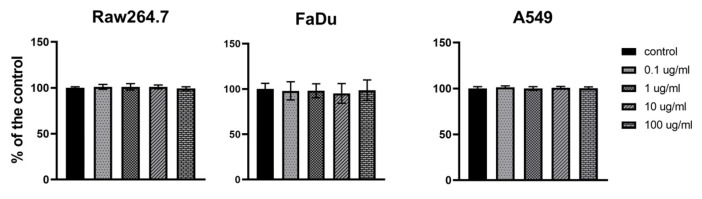
Cytotoxicity of PolaR testing on mammalian cells in vitro. FaDu (ATCC HTB-43), RAW 264.7 (ATCC TIB-71) and A549 (ATCC CCL-185) cell lines were treated with 0.1, 1, 10 or 100 µg/mL of PolaR. PBS of the same volume served as a control. Cells were incubated with PolaR for 48 h (ns *p* > 0.2).

The influence of PolaR on mammalian cell viability was tested via the MTT assay. FaDu (ATCC HTB-43) human pharynx squamous cell carcinoma, RAW 264.7 (ATCC TIB-71) mouse monocyte/macrophage and A549 (ATCC CCL-185) human lung adenocarcinoma were studied. Forty-eight hours of cell exposure to PolaR resulted in no harmful effects on tested cell lines. No toxic effect of PolaR was observed in any of the tested concentrations (0.1, 1, 10 or 100 µg) of the tested protein per ml (Figure 6).

## 4. Discussion

In this study, we used metagenomic data of phageomes extracted from human gastric mucosa biopsies to find a new candidate enzyme able to destroy *Rothia* bacteria. By searching for lysin-like sequences and identifying relevant bacterial hosts, we found a sequence coding for the PolaR enzyme that was further revealed as an active, anti-*Rothia* endolysin. This enzyme was studied in silico for its closely related homologs. While reference proteins similar to PolaR were generally found in genomes of *Rothia* species, closer inspection revealed that surrounding regions probably represent prophages. This finding would be consistent with multiple viral hits found in the IMG/VR database. Thus, we hypothesize that PolaR was found in a contig representing a lysogenic phage, probably released from the stomach-inhabiting *Rothia* species. This is in line with medical data of the patient whose stomach biopsy was the source for this metagenome, since the patient suffered from duodenal bulbitis and chronic gastritis that could be linked to bacterial dysbiosis, including overgrowth of *Rothia* spp. A 16S analysis of the bacterial part of this patients’ stomach microbiome revealed the presence of *Rothia mulcilaginosa* and *Rothia dentocariosa*.

PolaR production in the *E. coli* expression system followed by protein isolation and purification resulted in the protein clearly demonstrating antibacterial activity targeting *Rothia mucilaginosa* and *Rothia dentocariosa*. Notably, PolaR was active even against aggregated bacterial cells, acting against bacterial clumping and disrupting aggregates formed by *Rothia* cells, thus suggesting that biofilm formation (common in *Rothia* species) does not disturb PolaR antibacterial action. Since no harmful effects have been observed in mammalian cells treated with PolaR, we propose this enzyme as a new potential antibacterial agent that targets *Rothia*, an opportunistic pathogen causing severe infections particularly in immunocompromised patients. Difficult *Rothia* infections have been reported in neutropenic patients and pediatric cancer patients [48,49,50,51,52,53,54,55,56,57]. It can be associated with endocarditis, bacteremia, prosthetic infection and pneumonia [13,58,59,60,61,62,63,64] and it has been detected in the sputum of cystic fibrosis patients [12,15,16,17,18,65,66]. *Rothia*’s role as a pathogen is, however, probably underestimated, since it can be found in the human oral cavity and respiratory tract, and it is typically considered a part of the normal microbiota. Notably, *Rothia mucilaginosa* is able to release metabolites beneficial for *Pseudomonas aeruginosa* (a significant ESKAPE pathogen) metabolism, boosting its viability [16].

Our observations suggest that PolaR performs slower bacterial lysis compared to many other phage-derived lysins. On average, PolaR destroyed *Rothia*-formed aggregates in 2–4 h, and within the following few hours, the bacteria killing effect was observed. It is possible that for this enzyme, some cofactors such as metal ions or other conditions are able to induce faster performance. Particularly, some proteins with domains found in PolaR (amidase-2 and PGRP) are known to require metal ions—specifically zinc—for their efficient activity [67]. Additionally, some effects of the affinity tag type and localization in the recombinant form of PolaR cannot be excluded, and faster performance could potentially be achieved by its optimization. These observations should be a subject of further studies. 

This study also gives new insights into the biological role and characteristics of the stomach phageome, which is still an unexplored community. Even the bacterial part of the stomach microbiome has been underestimated for a long time, and the stomach mucosa was considered sterile or extremely poor in microbiota. This is due to the highly acidic environment of the stomach that limits the population of microorganisms, particularly compared to other parts of the gut such as the intestine. Additionally, to the best of our knowledge, no phage lysin has been found so far in this environment.

## 5. Conclusions

In silico analysis of metagenomic data provided a coding sequence for a candidate antibacterial enzyme, which was further positively verified for its ability to kill susceptible bacteria. PolaR is the first identified *Rothia mucilaginosa*- and *Rothia dentocariosa*-specific endolysin, demonstrating its dose-dependent antibacterial activity and its ability to destroy bacterial cells even when aggregated. This enzyme, though clearly active against bacteria, had no cytotoxic or antiproliferative effects on three different mammalian cell lines. Thus, PolaR may have a high potential to combat infections caused by *Rothia mucilaginosa* and *Rothia dentocariosa*; these include difficult-to-treat infections in immunocompromised or cystic fibrosis patients, e.g., pneumonia, endocarditis and septicemia. Other potential bacterial targets cannot be excluded, and they are a promising target for future studies.

PolaR is also the first antibacterial enzyme selected from the gastric mucosa phageome, which underlines the biological complexity and probably underestimated biological role of phageomes in human gastric mucosa.

## 6. Patents

P.M., K.D., A.W., J.B., S.B. are inventors of a patent (patent application no P.445254) owned by the Ludwik Hirszfeld Institute of Immunology and Experimental Therapy and Adam Mickiewicz University Poznań.

## Data Availability

The data presented in this study are available in the article or Supplementary Material. Phageome sequencing data used in presented analyses are available on NCBI as SRA and BioSample records linked to BioProject [68].

## Supplementary Materials

The following supporting information can be downloaded at: https://www.mdpi.com/article/10.3390/cells12151997/s1, File S1: Results of the bioinformatic analysis that led to the production of PolaR. The master xlsx spreadsheet includes summary statistics of the sequencing libraries’ trimming and assembly procedures, IMGVR search and quality metrics of different AlphaFold models. The raw output of the ColabFold pipeline is provided in the “PolaRAlphaFold” folder. Phylogenetic trees (and underlying alignments) of PolaR and reference proteins used to create Figure 1 are included as nexus, newick and phylip files. Figure S1: SDS-PAGE of the final preparation of PolaR., Figure S2: Antibacterial activity of PolaR against *Rothia aeria* PCM 2669 in time, Figure S3: Antibacterial activity of 150 µg/mL PolaR against *Rothia mucilaginosa* PCM 2415 (ATCC 25296) was tested over time in a turbidity reduction assay (TRA). Figure S4: Native-PAGE of PolaR preparation.
