# Supplementary figures and images for: New Phage-Derived Antibacterial Enzyme PolaR Targeting Rothia spp
Source: Cells. 2023 Aug 4;12(15):1997. doi: 10.3390/cells12151997 (PMC10417112; doi:10.3390/cells12151997)

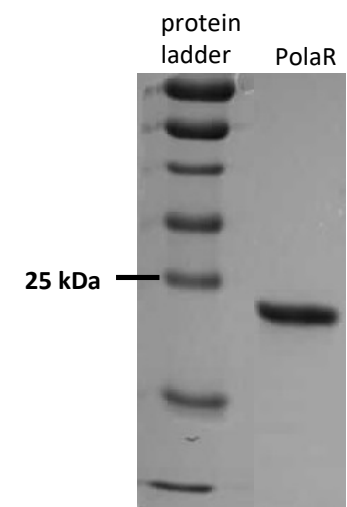

Supplement: Supplementary file 1 [file cells-12-01997-s001.zip › Figure S1.pdf]

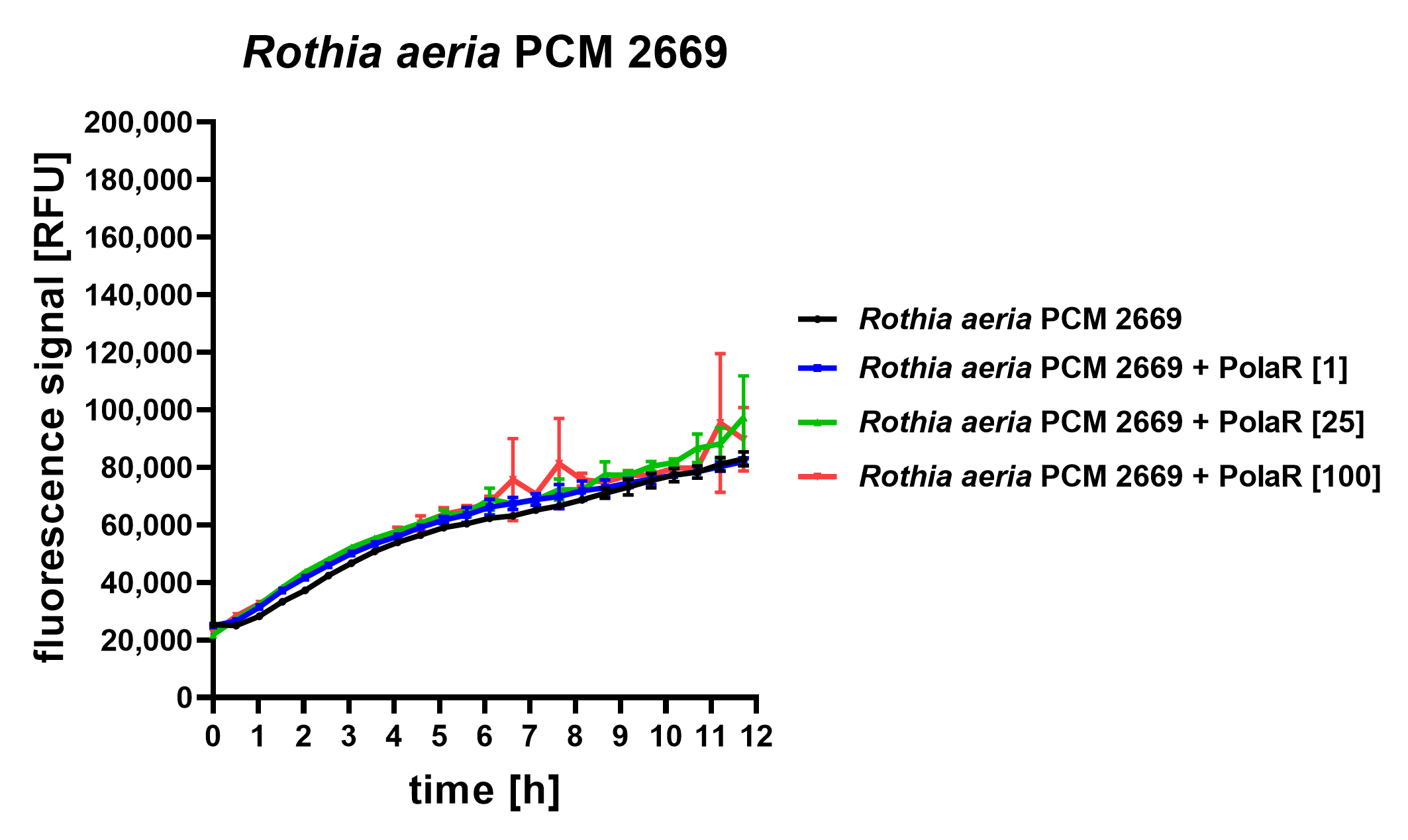

Supplement: Supplementary file 1 [file cells-12-01997-s001.zip › Figure S2.tif]

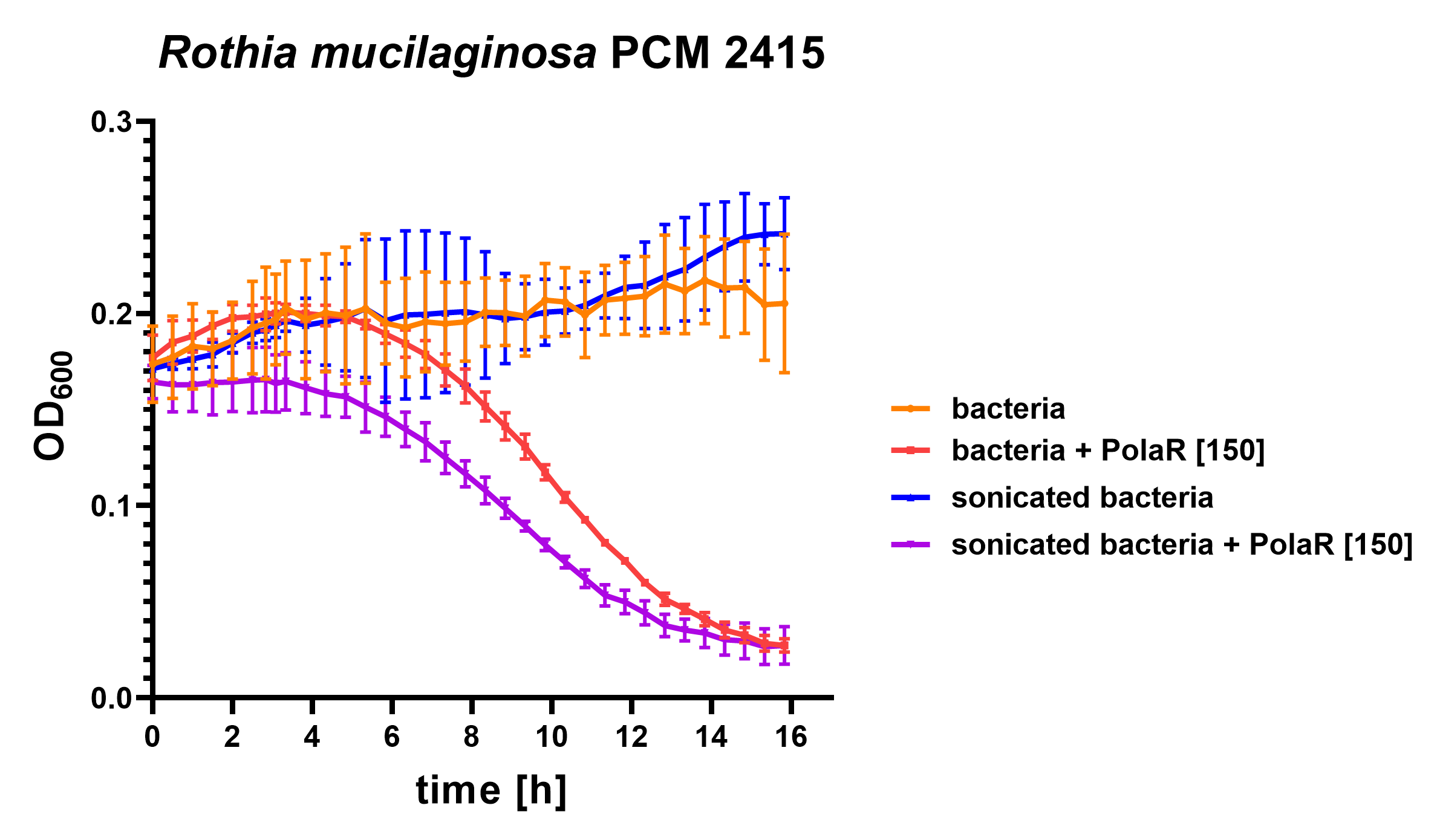

Supplement: Supplementary file 1 [file cells-12-01997-s001.zip › Figure S3.tif]

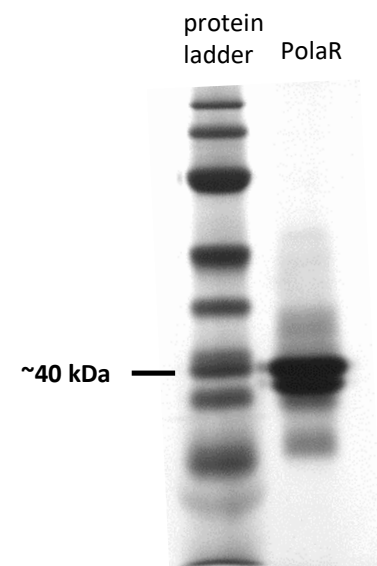

Supplement: Supplementary file 1 [file cells-12-01997-s001.zip › Figure S4.pdf]

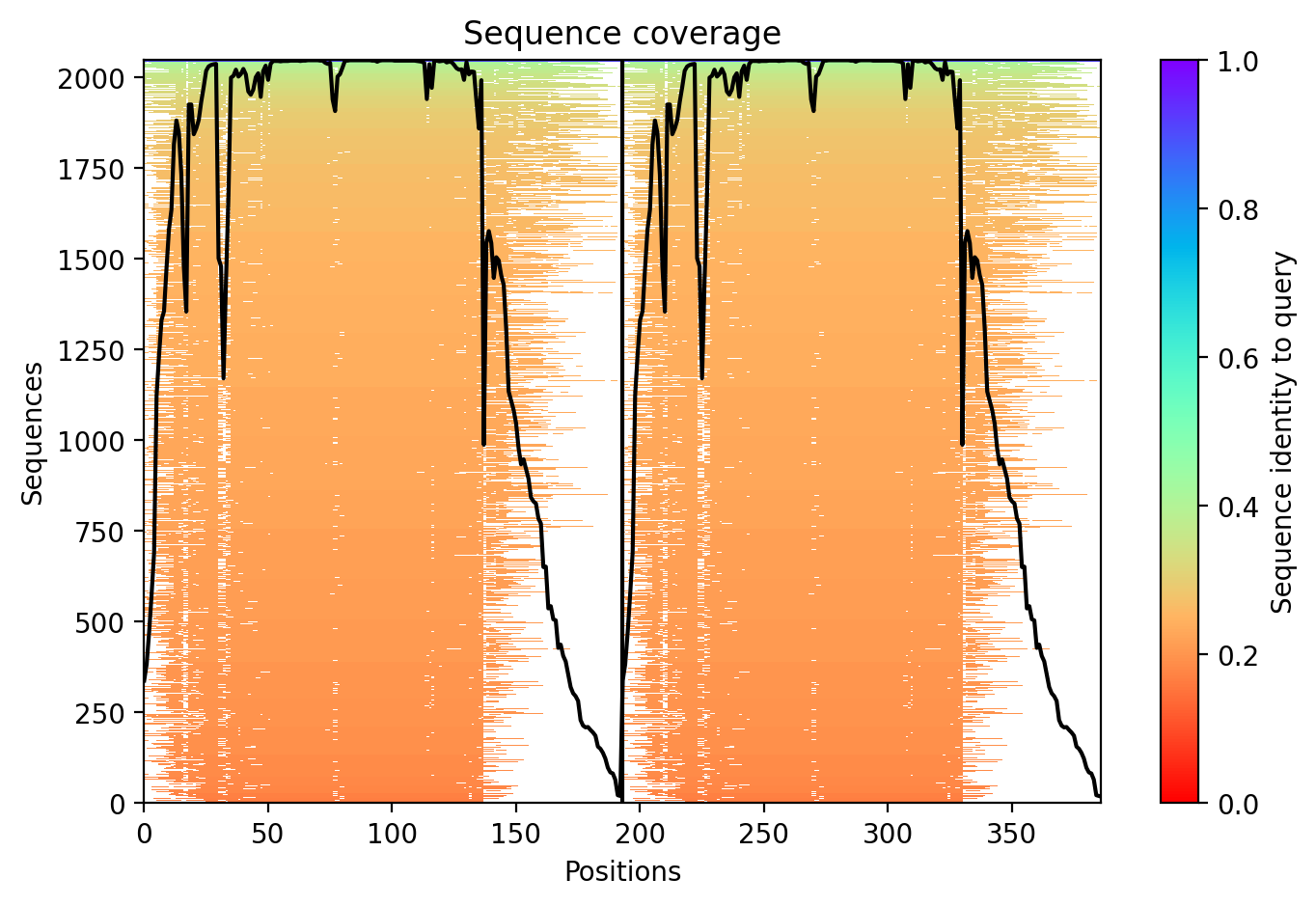

Supplement: Supplementary file 1 [file cells-12-01997-s001.zip › File S1/PolaR AlphaFold/dimer/Rothia_dimer_6e506_coverage.png]

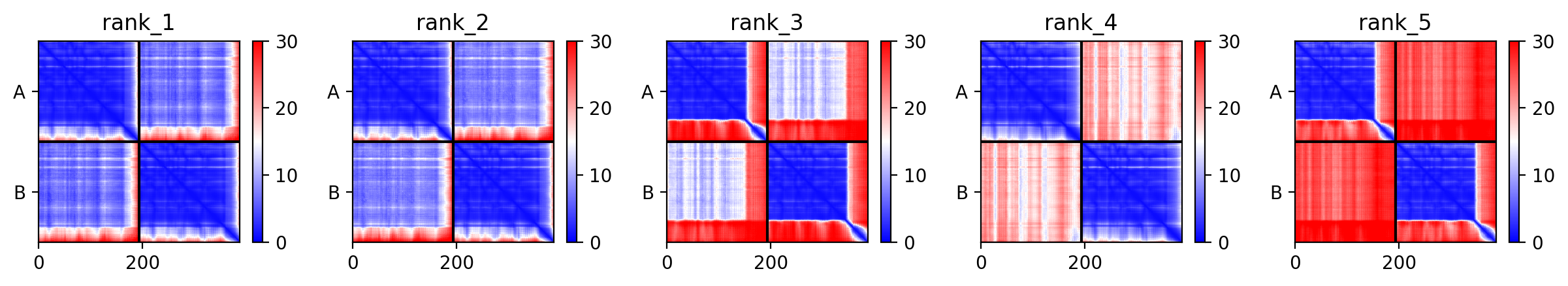

Supplement: Supplementary file 1 [file cells-12-01997-s001.zip › File S1/PolaR AlphaFold/dimer/Rothia_dimer_6e506_pae.png]

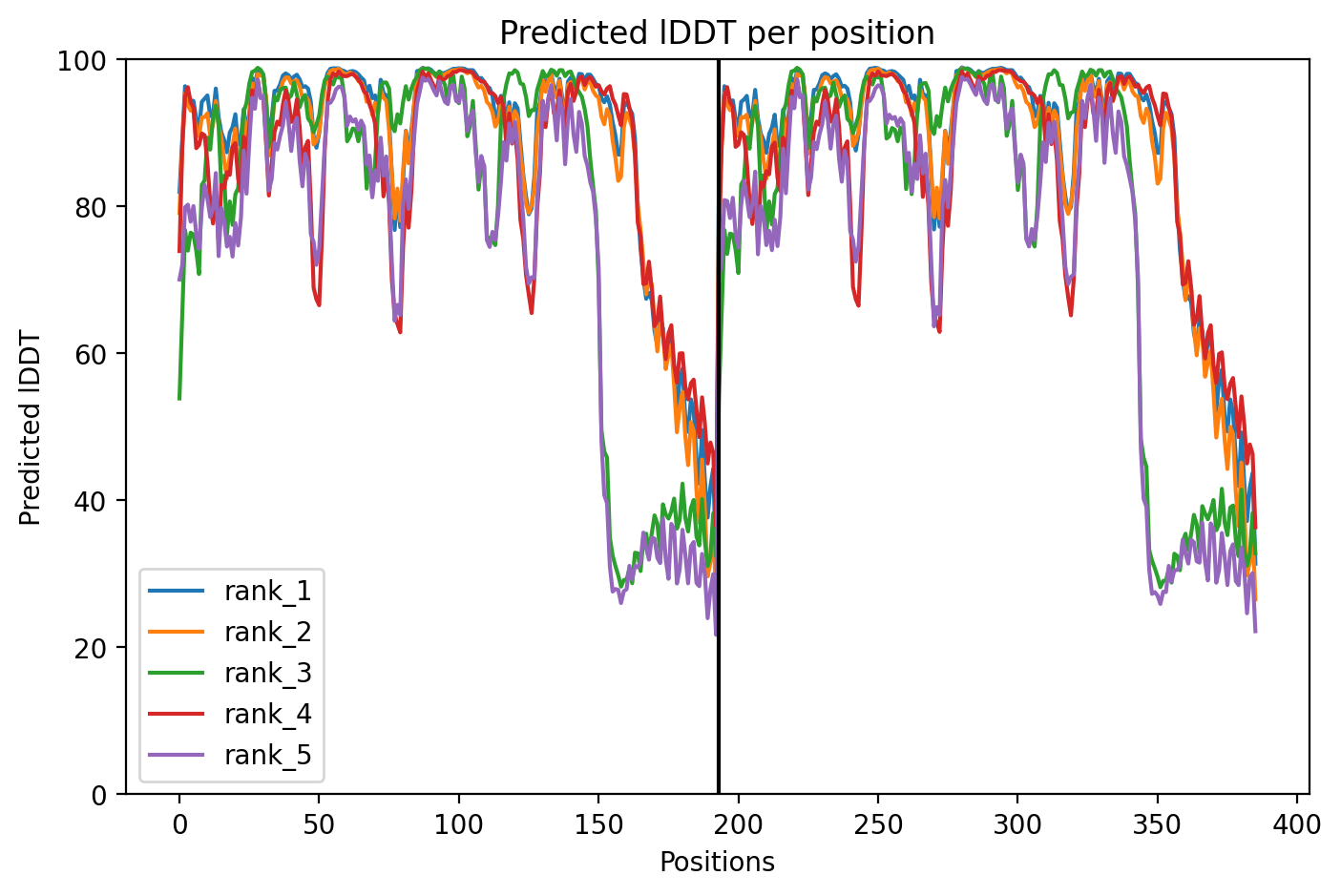

Supplement: Supplementary file 1 [file cells-12-01997-s001.zip › File S1/PolaR AlphaFold/dimer/Rothia_dimer_6e506_plddt.png]

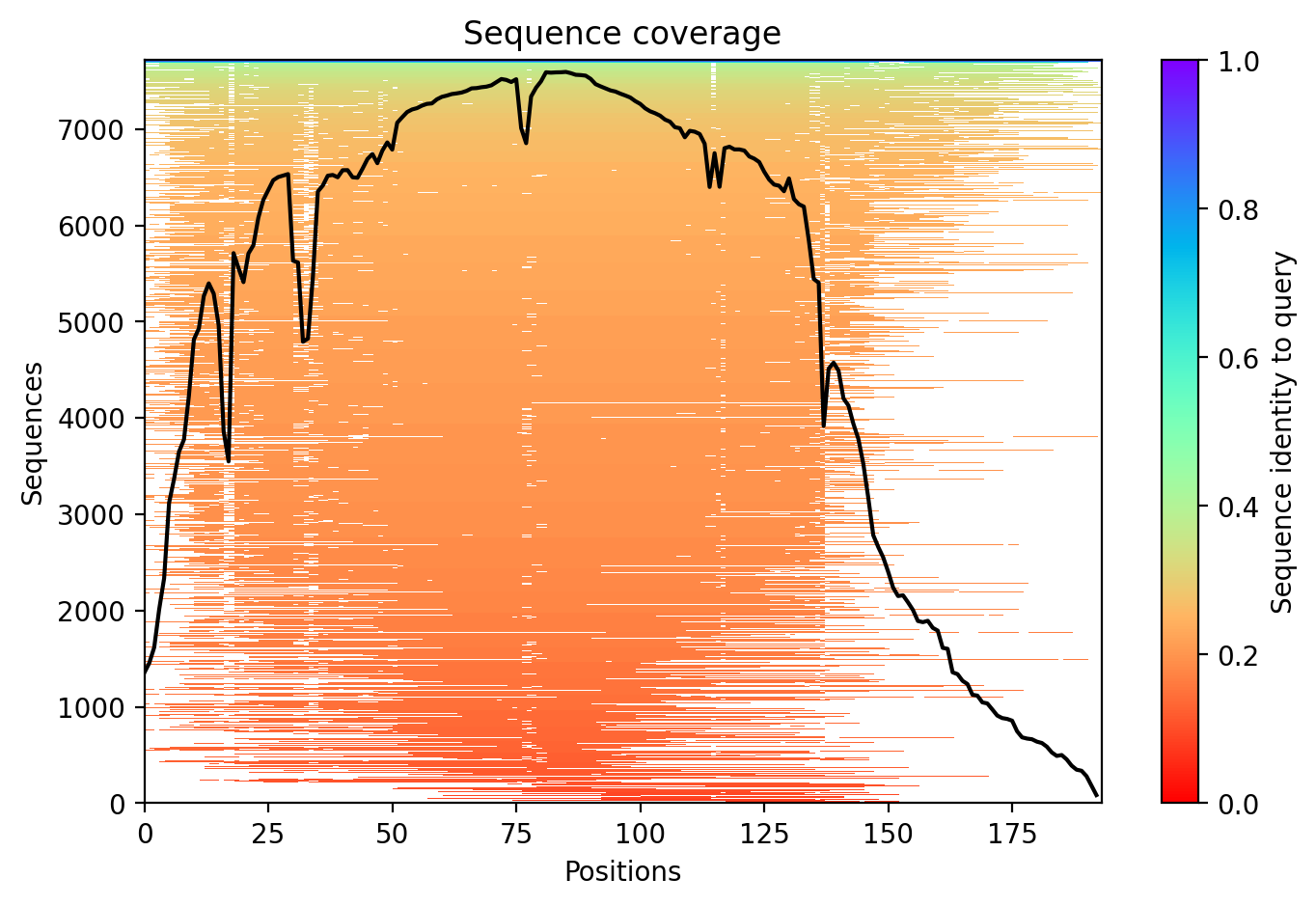

Supplement: Supplementary file 1 [file cells-12-01997-s001.zip › File S1/PolaR AlphaFold/monomer/Rothia_monomer_8696f_coverage.png]

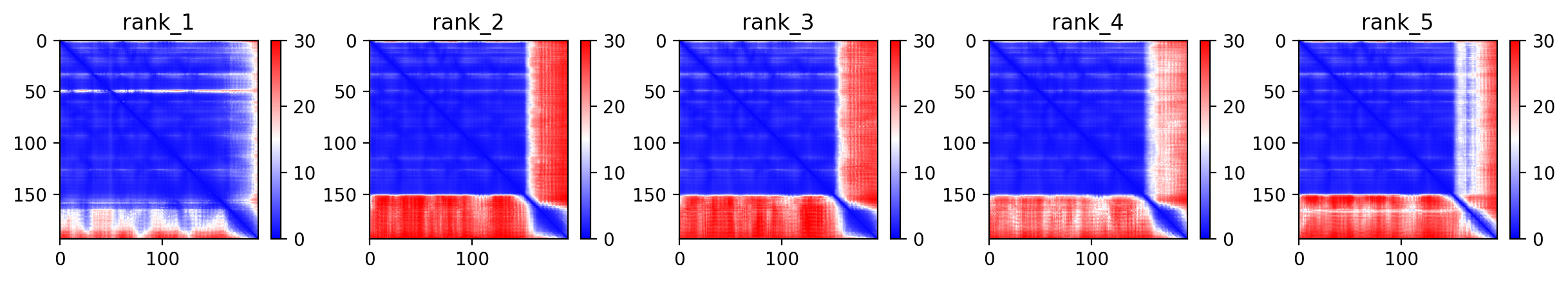

Supplement: Supplementary file 1 [file cells-12-01997-s001.zip › File S1/PolaR AlphaFold/monomer/Rothia_monomer_8696f_pae.png]

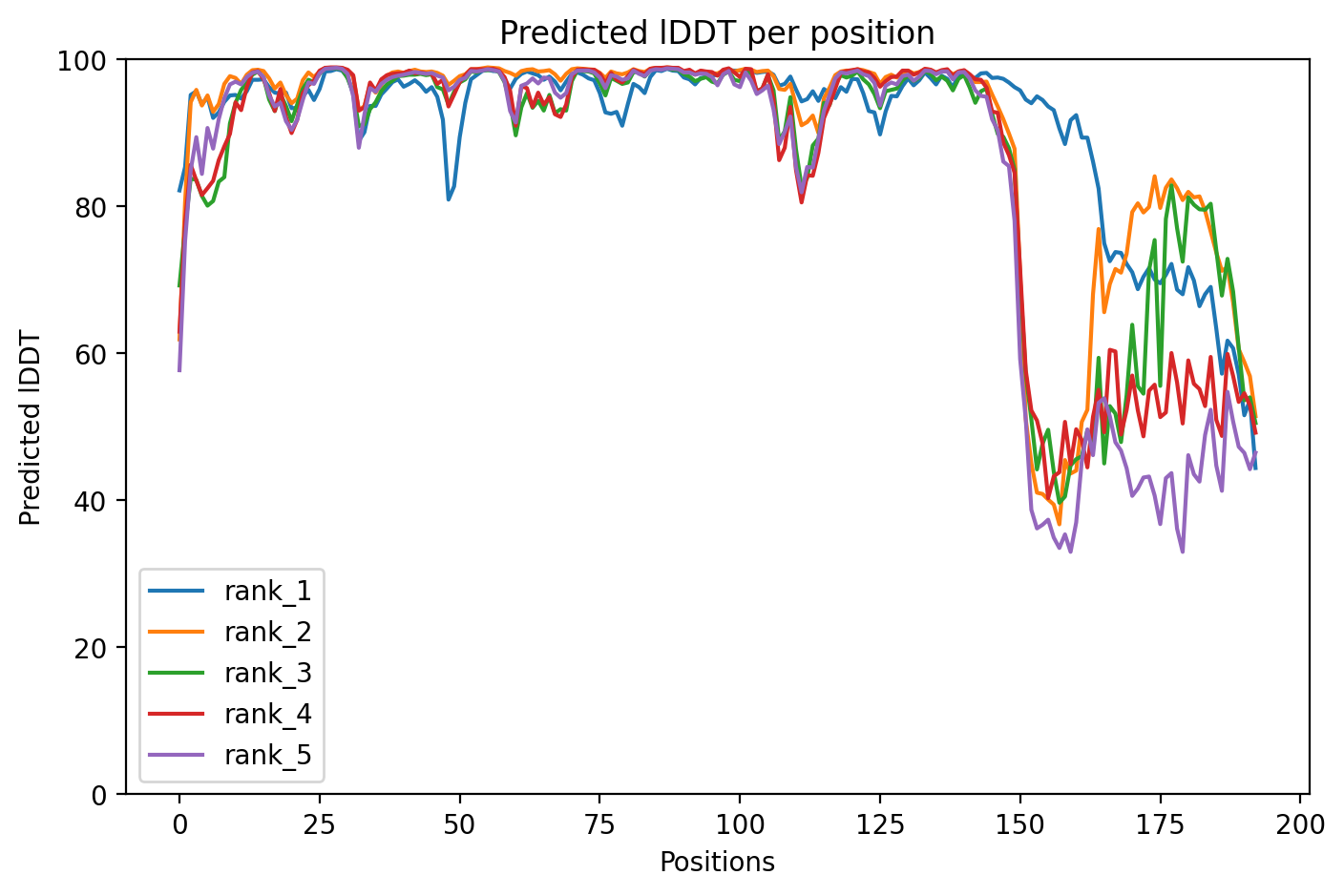

Supplement: Supplementary file 1 [file cells-12-01997-s001.zip › File S1/PolaR AlphaFold/monomer/Rothia_monomer_8696f_plddt.png]

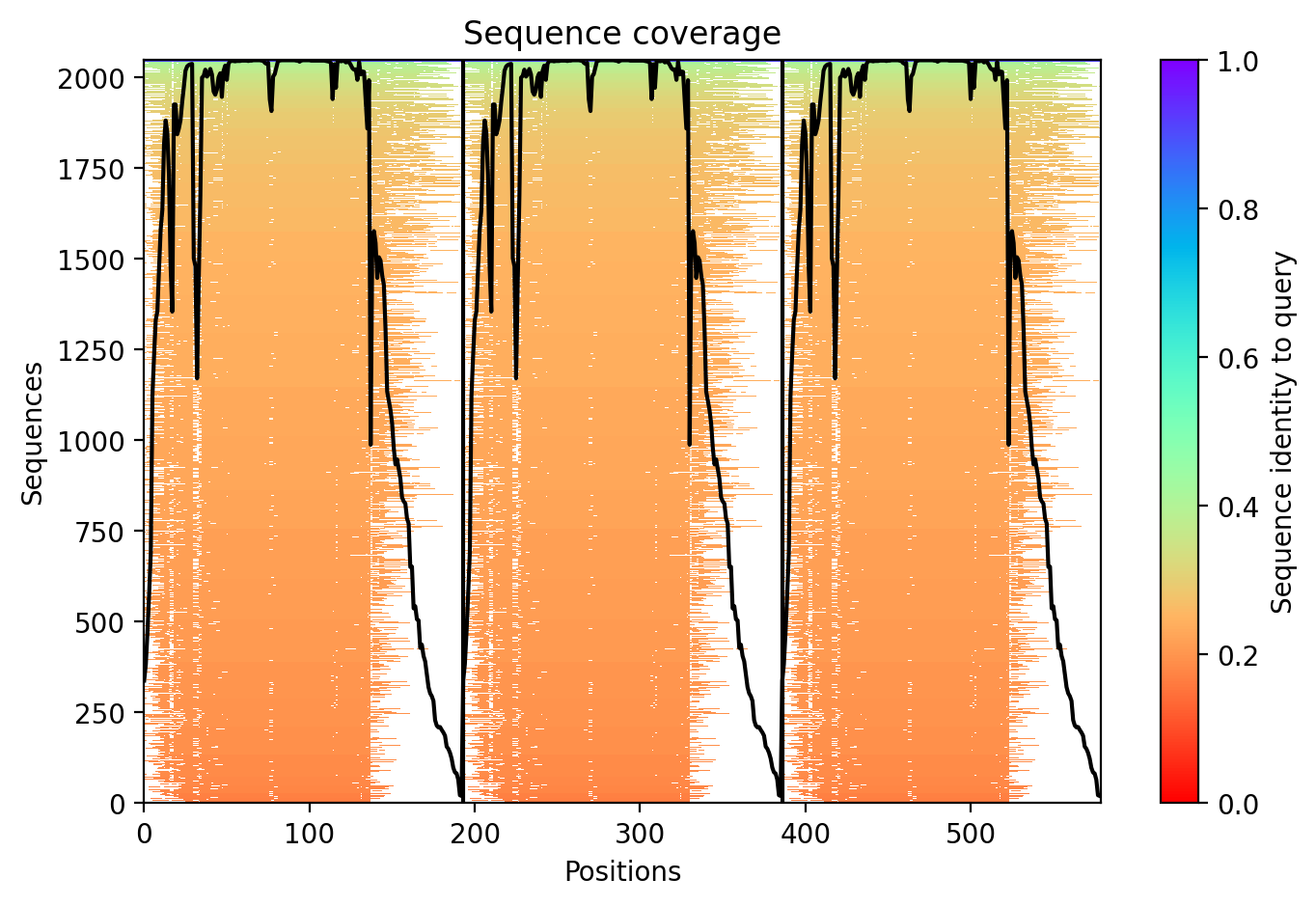

Supplement: Supplementary file 1 [file cells-12-01997-s001.zip › File S1/PolaR AlphaFold/trimer/trimer_rothia_f4755_coverage.png]

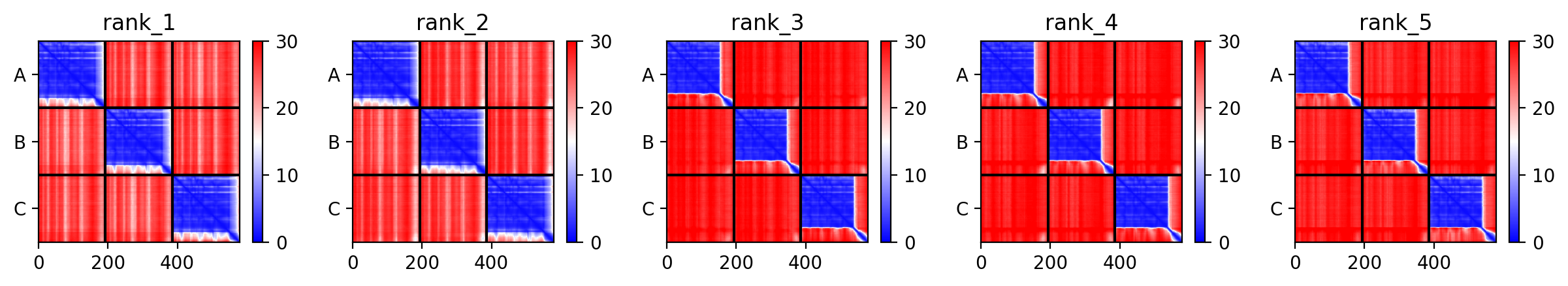

Supplement: Supplementary file 1 [file cells-12-01997-s001.zip › File S1/PolaR AlphaFold/trimer/trimer_rothia_f4755_pae.png]

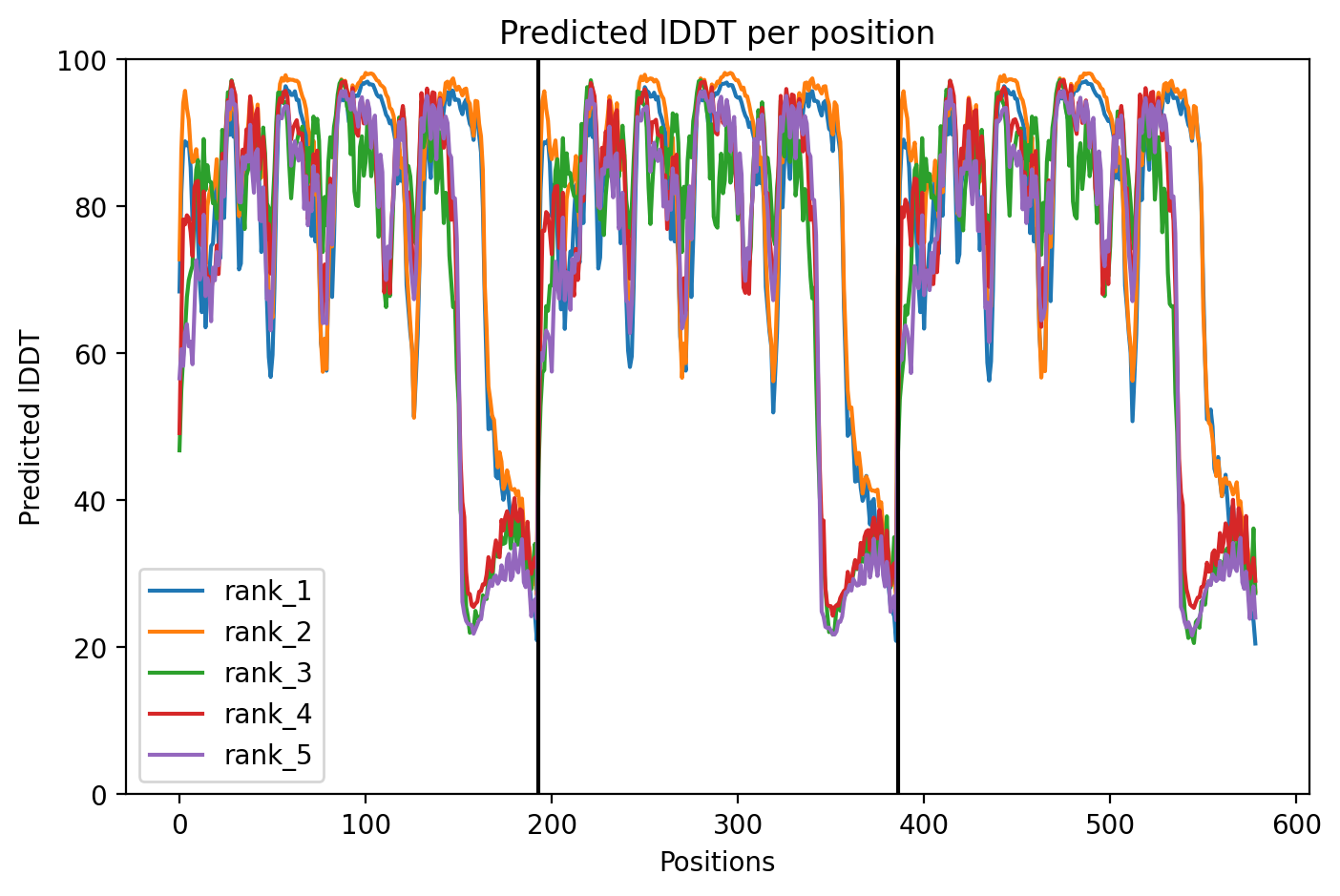

Supplement: Supplementary file 1 [file cells-12-01997-s001.zip › File S1/PolaR AlphaFold/trimer/trimer_rothia_f4755_plddt.png]
